# Autonomous restart of information floating and dynamic control of transmittable area

**DOI:** 10.1371/journal.pone.0341468

**Published:** 2026-01-29

**Authors:** Kazuyuki Miyakita, Daichi Meguro, Hiroshi Tamura, Keisuke Nakano

**Affiliations:** 1 Graduate School of Science and Technology, Niigata University, Niigata, Japan; 2 Faculty of Science and Engineering, Chuo University, Tokyo, Japan; National Marrow Donor Program, UNITED STATES OF AMERICA

## Abstract

Information floating (IF) is a method of delivering information to mobile nodes in a desired area while avoiding unnecessary communication and information dissemination by restricting direct wireless transmission to a transmittable area (TA). This restriction, however, also leads to the termination of IF, which is a longstanding problem that must be overcome. As a solution, methods have been developed to predetermine the optimal TA size based on environmental parameters such as node density. If the density changes over time, then the estimation of the density and the optimization of the TA must be repeated. Therefore, we previously proposed a method that guarantees that the IF never ends in principle, even if the node density changes over time, by dynamically controlling the TA size. However, this method is only applicable in a one-dimensional network. Here, we propose a method that guarantees, even in two-dimensional networks, that the IF never ends. To accomplish this, we introduce two key functions. The first autonomously restarts the IF even if it has temporarily terminated. The second function dynamically controls the TA size. We also highlight the necessity of introducing a lifetime for the TA generated by the dynamic control method if the density changes over time, and we improve the proposed method accordingly. We show the effectiveness of the proposed methods in terms of continuity and tracking performance through theoretical and simulation evaluations.

## 1 Introduction

If mobile nodes transmit information to other mobile nodes using direct wireless communication as they move, the information becomes spatially diffused. This type of information diffusion enables a system to deliver information to distant mobile nodes [1–6]. In this paper, we call this “epidemic communication.” Epidemic communication is an effective method in delay-tolerant networks (DTNs). However, this method has problems such as the spread of information to unrelated areas and the increase in unnecessary transmissions. One of the methods used to solve these problems is information floating (IF), which restricts the location of direct wireless transmissions to within the transmittable area (TA) [7–21]. In order to execute IF, each node must know its own location using a positioning technology such as GPS.

**Fig 1:**
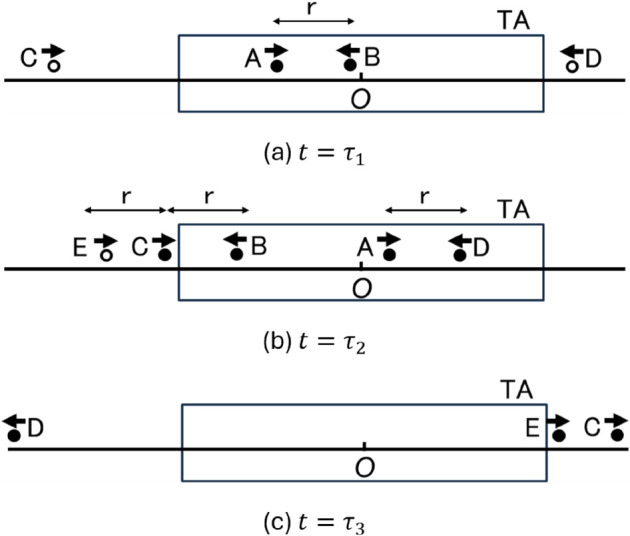
Example of IF. (a) $t=\tau_{1}$, (b) $t=\tau_{2}$, (c) $t=\tau_{3}$.

Fig 1 shows an example of IF. Suppose that IF delivers a message M.

M={data,TA}, (1)

where *data* is the data to be delivered and *TA* is the information on the position and size of the TA. In Fig 1, black and white circles indicate mobile nodes with and without M, respectively. The direct wireless communication range is *r*. The center of TA is the coordinate O. A is the first mobile node having message M. At time $t=\tau_{1}$, the distance between A and B becomes *r* and A is in TA, so A sends M to B. In the same manner, at time $t=\tau_{2}$, A and B send M to D and C, respectively. On the other hand, C does not send M to E, even though they are within each other’s communication range, because C is outside the TA. At time $t=\tau_{3}$, all nodes having M leave the TA. If no node having M enters the TA after this time, IF will never restart. This is the end of IF.

As described above, IF has a critical problem in that it ends if all nodes having information leave the TA while none enters the TA from outside. To overcome this problem, almost all papers on IFs have studied how to determine an appropriate TA size so that the IF continues for a sufficiently long time depending on the environmental parameters, in particular the node density. Note that this approach can only be applied if the node density is obtained by some method. Moreover, if the density changes over time, then the above estimation and optimization steps must be repeated.

One solution to achieve the proper adaptation is to dynamically control the TA. Consequently, various methods to accomplish this have been proposed. In one such method [18,20], each node having information sets the size of the TA based on its past experience of contacts with other nodes, and thus the node is expected to contact other nodes while in the TA. However, this is merely an expectation, and this method still has the same critical problem as using a fixed TA.

As an alternative, in a previous study [17] we proposed another dynamic control method for TA, where the IF never ends in principle. In this method, a node that has not sent information to other nodes within the TA expands the TA to its own position until it sends information to another node. Conversely, a node that has received information in the expanded TA shrinks the TA to its own position while moving toward the center of the TA, i.e., reference point O. By dynamically controlling the TA in this way, there is always at least one node having information within the TA, so it is guaranteed that the IF never ends. Although this method works well for a one-dimensional network, extending it to two-dimensional networks is not trivial, and there remain issues that must be overcome.

In addition, these dynamic controls have not been evaluated in situations where the density changes over time, although they are needed for these situations.

In this paper, we consider how to develop an IF method that never ends and can dynamically control a TA even in two-dimensional networks. As an extension to the two-dimensional case, we consider road networks spreading over a two-dimensional plane. In particular, we give attention to lattice networks. In this paper, we introduce the concept of autonomous restart (AR), which restarts the IF even if it has temporarily terminated. This restart process is carried out autonomously. In addition, the size of the TA is dynamically controlled using the information on the position where the AR function is executed.

The novel technical advances and contributions of this paper are as follows.

1. To achieve an IF method that never ends completely, even in two-dimensional networks, we introduce AR.
2. Simultaneously, we also propose a dynamic control method for TA. This allows the TA to be adjusted autonomously even if the density changes over time, and information can be delivered to many nodes while avoiding unnecessary transmissions.
3. We identify the following serious problem in the case of time-varying density. In general, dynamic TA control expands the TA as the density decreases. On the other hand, it is also designed to shrink the TA as the density increases. In actual situations, however, it is not easy to shrink the TA in the latter situation. Here, we present simulation results that illustrate this problem and propose a way to overcome this difficulty.
4. We present evaluations of the proposed methods as follows. First, we clarify how AR can restart a temporarily terminated IF through a theoretical analysis. Second, we demonstrate the effectiveness of the proposed methods through simulation. In particular, we show that they work well even in cases where the density changes drastically over time and that they significantly improve performance compared to traditional methods using a fixed TA.

To demonstrate the practical relevance of IF, we outline potential deployment scenarios and the practical challenges of its implementation. IF can be used to deliver local information to unspecified mobile nodes passing through a specific area, without the need for communication infrastructure. The following applications have been considered in the literature: distribution of local information or advertisements [7,12,14–16], distribution of traffic or accident information in vehicular ad hoc networks (VANETs) [7,11,14–16,19], distribution of emergency information during disasters [7,14,16,19,21], and applications to sensor networks [12]. Furthermore, the use of IF to provide available route information by virtually accumulating each vehicle’s travel history has been proposed [21]. In such applications, node density is likely to change over time; therefore, preventing IF from terminating completely in these situations is a significant practical challenge in its implementation.

The rest of this paper is organized as follows. In Sect 2, we first explain the proposed methods in a one-dimensional network to demonstrate our basic idea. In Sect 3, we extend the proposed methods to a two-dimensional road network that includes intersections. We also explain an additional operation for shrinking a TA in the proposed method, and we thus propose an improved method. In Sect 4, we evaluate the proposed methods using theoretical analysis and simulations. Sect 5 concludes this paper. For the convenience of readers, the acronyms used in this paper and their meanings are listed in Appendix A, and the theoretical computation of a key equation in Sect 4.2 is shown in Appendix B.

## 2 Proposed method in one-dimensional road model

In this section, we first consider a one-dimensional model to explain the basic idea of the proposed method. As in the previous work [17], let us suppose that reference point O is at the center of the TA.

### 2.1 Autonomous restart (AR-1d)

First, we explain the function of AR, which restarts an IF that has temporarily terminated. We refer to the AR used in a one-dimensional model as AR-1d. Note that AR-1d is different from the previous method [17] and is adopted with a view toward its extension to two dimensions.

In AR-1d, we add a flag to message M as follows.

M={data,TA,flag,O}. (2)

*flag* takes the value 1 or 0, and it is used to determine whether the node has the role of restarting IF. A node having M with *flag* = 1 has to restart IF. To do this, this node continues searching for approaching nodes and sends M with *flag* = 1 to the first contacted node after passing O, even if this node is outside the TA. On the other hand, a node having M with *flag* = 0 executes normal IF.

**Fig 2:**
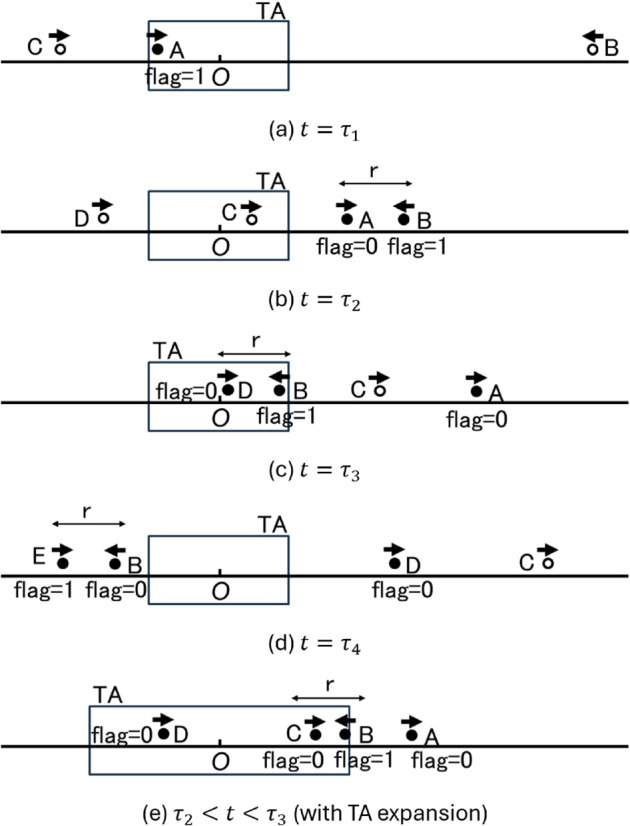
Example of AR-1d. (a) $t=\tau_{1}$, (b) $t=\tau_{2}$, (c) $t=\tau_{3}$, (d) $t=\tau_{4}$, (e) $\tau_{2}<t<\tau_{3}$ (with TA expansion).

We explain the operation of AR-1d using the example in Fig 2. A is the node that starts the IF. Therefore, A is the only node having M with *flag* = 1 at the initial moment $t=\tau_{1}$. Between $\tau_{1}$ and $\tau_{2}$, A does not contact any node; therefore, at time $t=\tau_{2}$, A has left TA and the IF has temporarily terminated. However, because A has M with *flag* = 1, A sends M with *flag* = 1 to B, which is approaching O, even though A is outside the TA, and then sets its *flag* to 0. At time $t=\tau_{3}$, B enters TA and restarts IF, and M is sent from B to D. Here, since B has not yet passed O, B does not send M with *flag* = 1 to C but instead sends M with *flag* = 0. Then, at time $t=\tau_{4}$, B sends M with *flag* = 1 to E, since E is the first contacted node after B has passed O.

As seen from these procedures, a node having M with *flag* = 1 always exists in the system, and this node is always moving toward O. Therefore, even if the IF has temporarily terminated, it will always restart, and thus the IF never terminates completely. Also, in AR-1d, the number of nodes having M with *flag* = 1 does not increase, in order to avoid increasing useless transmissions. As a result, there is always only one node having M with *flag* = 1 in the system.

Note that to execute AR-1d, each node must know the moving directions of itself and its neighbors, as well as its own position. Therefore, in AR-1d, it is assumed that each node measures its own moving direction from the time variation of its location information and exchanges this information with its neighbors.

### 2.2 Dynamic control of TA (DC-1d)

In the example shown in Fig 2, AR-1d can prevent the complete termination of IF, but there are nodes, such as node C, that cannot receive information by passing through the TA during the temporal termination of IF. To reduce the occurrence of such nodes, we consider a method of dynamically controlling the TA based on the location where M with *flag* = 1 is transmitted. For example, in Fig 2, if the TA is expanded to the location of A at $t=\tau_{2}$, C will be able to receive M from B, as shown in Fig 2e. We call this method DC-1d.

**Fig 3:**
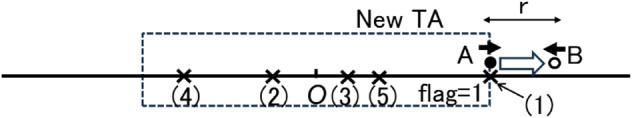
Example of DC-1d, where n=5. (*i*) indicates the *i*th most recent position where M with *flag* = 1 was transmitted.

As mentioned above, in DC-1d, when M with *flag* = 1 is sent in the operation of AR-1d, TA is updated to include the location where M with *flag* = 1 was sent. Here, if the TA is updated to include only the latest location where M with *flag* = 1 was sent, the TA will be updated significantly and frequently due to the randomness of the node locations, and thus the system will become unstable. To prevent this, each node stores a history of the locations where M with *flag* = 1 was sent in the past *n* times and then updates the TA to include all of these *n* locations, where *n* is a predetermined positive integer. Specifically, we determine the new TA as the region to the furthest point in the history of *n* positions, while making it symmetrical with O as the center (Fig 3). Such a method of determining TA facilitates an easy extension to two dimensions.

To perform the above algorithm, in DC-1d we add new parameters to message M as follows.

$$M=\{data,TA,flag,O,t_{TA},\{h_{1},h_{2},\cdots ,h_{n}\}\},$$ (3)

where *t*_*TA*_ is the time when the TA information was updated and $\left\{h_{1},h_{2},\cdots ,h_{n}\right\}$ is the history of the most recent *n* positions where M with *flag* = 1 was transmitted. When two nodes having different *TA* values contact each other, they compare the *t*_*TA*_ values and update the *TA* and *t*_*TA*_ values to those of the node having the more recent *t*_*TA*_ value.

## 3 Extension to two-dimensional road model with intersections

In this section, we extend AR-1d and DC-1d to two dimensions. As with the one-dimensional model, we consider a reference point O in the two-dimensional model, and we assume that this reference point is at the center of the TA. Unlike the case of the one-dimensional model, the two-dimensional model has some nodes that do not pass through O. Therefore, the purpose of AR in the two-dimensional model is to deliver information to the nodes that pass through O.

In the one-dimensional model, the node receiving M with *flag* = 1 always goes to O immediately. On the other hand, in the two-dimensional model, such a node does not always go to O but may move in a different direction from O, since it may turn before reaching O. In this paper, we assume that nodes move along roads without detours. Therefore, once a node has moved away from O, it never returns to O, and another node needs to return the M with *flag* = 1 to O.

**Fig 4:**
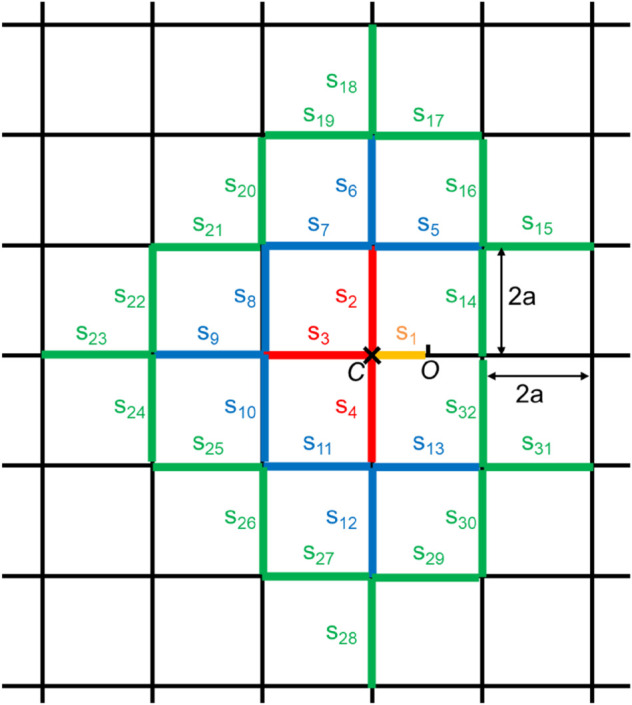
Lattice road network.

For example, consider a lattice road network as shown in Fig 4, where *s*_1_, *s*_2_, *s*_3_, ⋯ are road segments. O is assumed to be at the center of a road segment. If node A having M with *flag* = 1 cannot send M to an approaching node in *s*_1_, then A enters *s*_2_, *s*_3_, or *s*_4_ while keeping M with *flag* = 1. Suppose that A enters *s*_2_ and sends M with *flag* = 1 to another node B. If B enters *s*_1_, M with *flag* = 1 arrives at O within a short time. Otherwise, B enters *s*_3_ or *s*_4_, and M with *flag* = 1 moves away from O. Such behavior in lattice networks is different from that in AR-1d. Hence, we need to consider the effects of this kind of behavior toward achieving AR in two-dimensional networks.

### 3.1 Autonomous restart (AR-2d)

To overcome the above difficulty in the two-dimensional case, we extend AR-1d and DC-1d to AR-2d and DC-2d, respectively.

AR-2d is basically the same as AR-1d, and a node having M with *flag* = 1 sends it to the first contacted node after passing O. However, in AR-2d, if a node having M with *flag* = 1 turns in a direction away from O at an intersection before reaching O, it sends M with *flag* = 1 to the first contacted node after making this turn.

As shown in the above explanation, we must evaluate how long it is necessary to wait for an M with *flag* = 1 to return to O after the previous M with *flag* = 1 has left O. Therefore, we discuss this issue using the theoretical analysis in Sect 4.2.

### 3.2 Dynamic control of TA (DC-2d)

DC-2d is basically the same as DC-1d, but the way of determining the TA is different from that for DC-1d, since it corresponds to a road network with intersections. In DC-2d, TA is determined as the region from O to the furthest point in the *n* most recent positions where M with *flag* = 1 was transmitted, while making it symmetrical with O as the center. Specifically, if *d*_*i*_ is the distance along the road network from O to the *i*th latest position where M with *flag* = 1 was transmitted, then the TA is every region within distance $\max\{d_{1},d_{2},\cdots ,d_{n}\}$ along the road network from O.

### 3.3 Additional operation for shrinking TA and improving DC-2d: Proposal of DC-2d-i

In this subsection, we explain the additional operation done for the shrinkage of TA in the proposed method and further propose an improved version of DC-2d.

**Fig 5:**
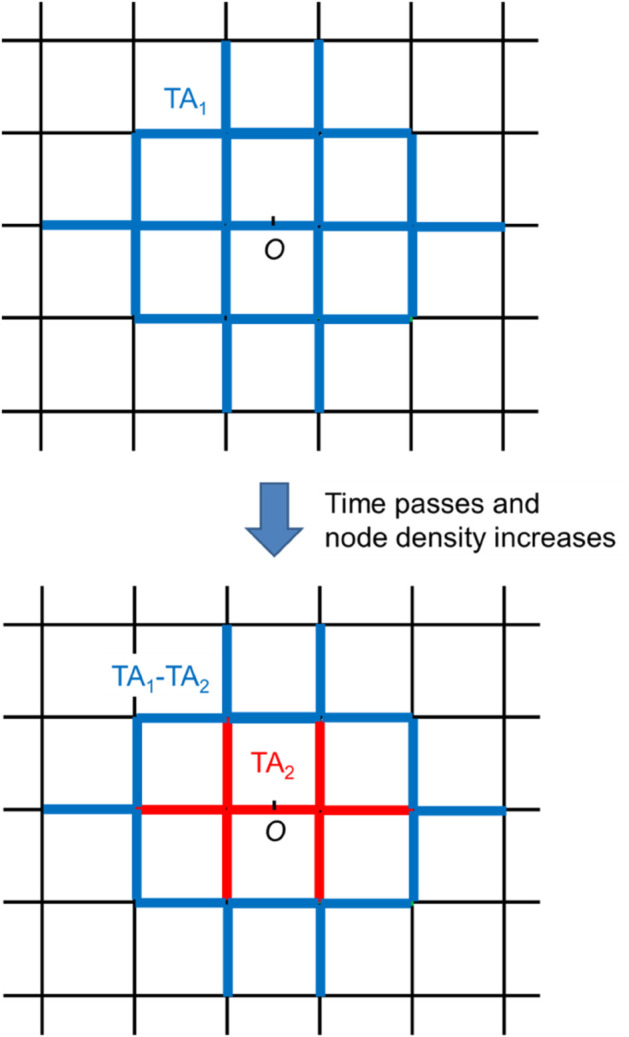
Problem where TA does not shrink.

Suppose that at time $t=\tau_{1}$, M with *flag* = 1 is transmitted and the TA is set to *TA*_1_ by the function of DC-2d. After that, at time $t=\tau_{2}$ ($\tau_{2}>\tau_{1}$), M with *flag* = 1 is transmitted, and the TA size is set to *TA*_2_. Here, we assume that the node density increases between $\tau_{1}$ and $\tau_{2}$, and as a result, *TA*_2_ becomes smaller than *TA*_1_. An example is shown in Fig 5. This *TA*_2_ is the new TA, but since M, which includes information about *TA*_2_, is basically only transmitted within *TA*_2_, the information that the TA is updated to *TA*_2_ is not delivered outside *TA*_2_. In other words, in region *TA*_1_ − *TA*_2_, *TA*_1_ continues to be used. However, this is undesirable because it increases unnecessary transmissions.

To avoid the above problem, we improve DC-2d so that the TA information is reset after a certain lifetime since the TA was made. We call this improved method DC-2d-i. Here, resetting the TA information means setting the TA size to 0, i.e., making the TA only O. In this paper, the lifetime is determined dynamically in the same way as the TA size. If the lifetime is too short, all TA information in the service area is reset before the next TA is made, resulting in many nodes not receiving M. With this in mind, we set the lifetime to approximately the time until the next TA is made, as follows.

Assume that at time $t=\tau_{0}$, M with *flag* = 1 is transmitted and the latest TA information *TA*_0_ is made. Let *d*_0_ be the distance from O to the edge of *TA*_0_ (see explanation in Sect 3.2, from which we derive $d_{0}=\max\{d_{1},d_{2},\cdots ,d_{n}\}$). The node receiving M with *flag* = 1 at $t=\tau_{0}$ will make the next TA, and it is expected that this will be done, at the latest, before this node leaves *TA*_0_. This time is approximately $2d_{0}/v$ because the distance from one edge of *TA*_0_ to the other is 2*d*_0_. Here, *v* is the velocity of each node, and this is assumed to be known or estimable. As a result, in DC-2d-i, the TA information *TA*_0_ made at $t=\tau_{0}$ is reset at $t=\tau_{0}+2d_{0}/v$. Then, if the node receives TA information made after $t=\tau_{0}$ or makes a new TA by receiving M with *flag* = 1, it updates the TA information.

In Sect 4.3, which presents simulation results, we discuss this phenomenon and the effectiveness of the improved method.

## 4 Performance evaluation

### 4.1 Assumptions

**Fig 6:**
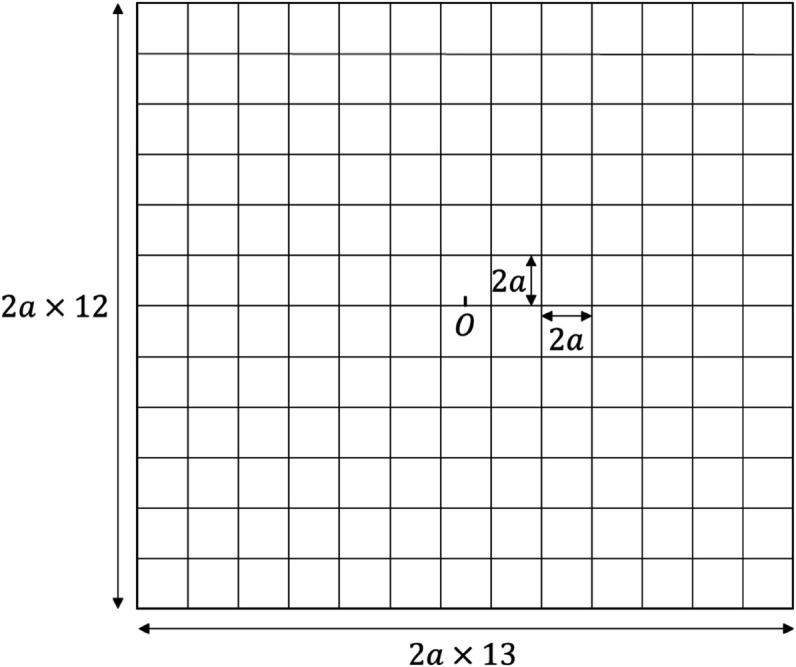
Simulation model.

In this section, we evaluate the proposed method. As a model of a two-dimensional road network, we use the lattice model shown in Fig 6. This is a 13 × 12 lattice structure, where each side of the lattice has a length of 2*a* = 500 m. Mobile nodes enter the lattice network from each of the roads at the edge of the lattice network according to a Poisson process with density *λ*. Each mobile node moves without detour as follows. There are four groups of nodes. For Groups 1, 2, 3, and 4, a node moves to the right or up, left or up, right or down, and left or down, respectively. The node densities of these four groups are the same. For Group *i* and intersection *j*, a node turns with probability *p*_*turn*,*i*,*j*_ and goes straight with probability 1 − *p*_*turn*,*i*,*j*_ after passing the intersection, where *p*_*turn*,*i*,*j*_ is a predetermined value. At the initial time of the simulation, the first node having M with *flag* = 1 leaves O and then moves to the left.

As described in the preceding section, the proposed method attempts to overcome the problem of M not returning to the TA. The achievement of this goal should be considered in the evaluation of the proposed method. In the above mobility model, mobile nodes move along the road network without detours and never turn back; therefore, this model is suitable for our objective because mobile nodes never return to the TA once they leave it. Note that if we use the random waypoint (RWP) mobility model, which is often used to evaluate the performance of mobile networks, mobile nodes often turn back to the previous area. Therefore, the RWP model is not suitable for our objective.

Regarding communication and node movement, to initially evaluate the fundamental performance of the proposed method, we consider the following simplified assumptions (Basic Factors BF1 to BF5).

- **BF1:** Communication is possible if and only if the nodes are within a constant communication range *r* = 100 m.
- **BF2:** There is no shadowing effect, and communication always succeeds when the straight-line distance between the nodes is within *r*.
- **BF3:** Each node moves at a constant velocity v=10 m/s  = 36 km/h.
- **BF4:** For all node groups *i* and all intersections *j*, *p*_*turn*,*i*,*j*_ is constant and is 2/3. As can be easily seen, this causes the nodes on the road to obey a Poisson process with an even density of *λ* in each direction on each road segment when looking at a snapshot.
- **BF5:** There is no GPS error, and all nodes can always accurately determine their own position.

The theoretical analysis and the simulation results with these simplified assumptions are shown in Sects 4.2 and 4.3, respectively.

In addition, to investigate the effects of variation and randomness in real situations, we also consider the following additional assumptions (Additional Factors AF1 to AF5).

**Fig 7:**
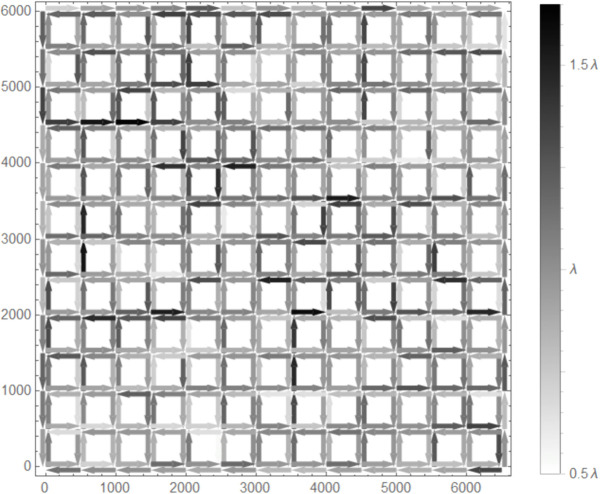
Uneven node density.

- **AF1:** A probabilistic link error model is introduced [22]. This model assumes that the communication success rate is $H(x)=\exp\{-(\frac{x}{r}{)}^{\eta }\}$, where *x* is the distance between two nodes. *η* is the path loss exponent, and we set it to η=2. Each node having information attempts transmission every Δt=1 s, and for each attempt the success or failure of communication is determined by the above probability.
- **AF2:** A shadowing effect is introduced. We assume that all areas outside the roads in the lattice road network shown in Fig 6 are obstructed by buildings. Consequently, communication between diagonal directions is impossible, and only nodes moving along the same road can communicate with each other.
- **AF3:** Variation in node speed is considered. There are two types of nodes, moving at speeds $v_{1}=7.5$ m/s and $v_{2}=15$ m/s, coexisting in equal traffic volumes. This scenario assumes all road segments are dual carriageways, where overtaking the vehicle in front is possible. However, to simplify the simulation, lane widths are not considered. Note that the mean time required for each node to travel a unit distance *x* [m] is $(x/v_{1}+x/v_{2})/2=x/10$ s, which is identical to the mean time for BF3, namely x/v=x/10 s.
- **AF4:** An uneven node density model is considered. To create this, we set *p*_*turn*,*i*,*j*_ to a random value (a uniform random number between 1/4 and 3/4) for all *i* and *j*. As a result, the density distribution becomes uneven, as shown in Fig 7.
- **AF5:** A GPS error model is introduced [23]. This model assigns errors following independent normal distributions (mean 0, standard deviation *σ*) in both x-axis and y-axis directions. Here, we set σ=5 m. Each node having information determines whether it is within the TA and its direction of movement every Δt=1 s, with the GPS error determined by the above distribution at each time.

The simulation results with these additional assumptions are given in Sect 4.4.

### 4.2 Evaluation of AR-2d’s ability to restart IF by theoretical analysis

Before showing the simulation results of the proposed method, we evaluate AR-2d’s ability to restart IF using a theoretical analysis. To do this, we theoretically compute how far away from O the M with *flag* = 1 moves before returning to O. In addition, by using the derived formula, we show that the temporarily terminated IF is always restarted, except in the case of extremely low density. In this subsection, we assume only BF1 to BF5. The numerical results of the formula are also used in the discussion on the simulation results in Sect 4.3.

Let *A*_0_, *A*_1_, *A*_2_, and *A*_3_ be the events where M with *flag* = 1 that departed from the left side of O returns to O without leaving the orange, red, blue, and green road segments in Fig 4, respectively. In general, for i≥0, *A*_*i*_ is the event where M with *flag* = 1 that departed from the left side of O returns to O without going further than *i* road segments from intersection C. We compute and evaluate $\mathrm{Pr}(A_{i})$, where Pr(·) is the probability that event ⋅ occurs. Of course, since this is a symmetric model, we can also evaluate M with *flag* = 1 that departed from the right side of O in the same way using $\mathrm{Pr}(A_{i})$. Theoretical computation of $\mathrm{Pr}(A_{i})$ is given in Appendix B.

**Fig 8:**
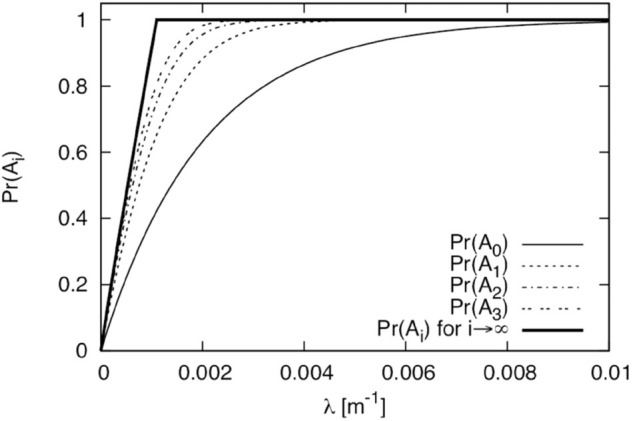
Characteristics of $\mathrm{Pr}(A_{i})$.

The numerical results for $\mathrm{Pr}(A_{i})$ are shown in Fig 8. The horizontal axis is *λ*. From this figure, we can see that $\mathrm{Pr}(A_{0})$ approaches 1 only when the density is significantly large (roughly greater than 0.01 m^−1^). Consequently, at such a density, M with *flag* = 1 that departed from the left side of O returns to O without leaving the orange road segment. Furthermore, it returns to O without leaving the red, blue, and green road segments when *λ* is greater than 0.004 m^−1^, 0.0025 m^−1^, and 0.002 m^−1^, respectively. From these results, in the operation of DC-2d and DC-2d-i, when the values of *λ* are approximately 0.01, 0.004, 0.0025, and 0.002 m^−1^, the TA is expected to be the regions within the orange, red, blue, and green road segments, respectively.

As shown in Appendix B, we can also compute the value of $\mathrm{Pr}(A_{i})$ for i→∞, and this value is 1 if λ≥log3/(4a)≈0.001099 m^−1^. This theoretically guarantees that if the service area is sufficiently large, M with *flag* = 1 always returns to O, except in the case where the node density is extremely small.

### 4.3 How successfully the proposed method restarts IF and dynamically controls TA: Simulation results

Here, we show how successfully the proposed method restarts IF and dynamically controls TA by using computer simulations. This subsection also assumes only BF1 to BF5. We consider the situations where the node density changes over time with the following two patterns of density change.

- Pattern 1:
  $$\lambda =\left\{ \begin{array}{cc} 0.004{\text{ m}}^{-1}, & 0\leq t<10000\text{ s},\\ 0.002{\text{ m}}^{-1}, & 10000\text{ s }\leq t<20000\text{ s},\\ 0.004{\text{ m}}^{-1}, & 20000\text{ s }\leq t\leq 40000\text{ s}. \end{array}\right.$$ (4)
- Pattern 2 (more significant density changes than Pattern 1):
  $$\lambda =\left\{ \begin{array}{cc} 0.01{\text{ m}}^{-1}, & 0\leq t<10000\text{ s},\\ 0.001{\text{ m}}^{-1}, & 10000\text{ s }\leq t<20000\text{ s},\\ 0.01{\text{ m}}^{-1}, & 20000\text{ s }\leq t\leq 40000\text{ s}. \end{array}\right.$$ (5)

We evaluate the following seven metrics. The first is the mean of the time until the IF is completely finished, namely the time until all nodes having information leave the service area, denoted by *E*(*T*_*f*_). The second is the mean of the size of *TA* (i.e., the distance from O to the edge of TA along the road network) for all nodes having M at each time *t*. This verifies that the TA information is updated appropriately. Note that when calculating this metric for DC-2d-i, we exclude the nodes whose TA size is 0 due to resetting the TA information. The third is the ratio that nodes passing through O receive M before leaving the service area, denoted by *p*_*rec*_. Since the purpose of IF in the two-dimensional model is to deliver information to nodes passing O as mentioned above, we use this metric as the information reception rate. The fourth is *R*_*useless*_:

$$R_{useless}=\frac{\text{number of times }\text{data}\text{ in M is sent to a node that does not pass through O}}{\text{number of times }\text{data}\text{ in M is sent to a node that passes through O}}.$$ (6)

*R*_*useless*_ indicates how many useless transmissions are made to complete one necessary transmission, and it is thus used to evaluate the number of useless transmissions. The fifth, sixth, and seventh metrics are the numbers of transmissions in M of *data*, TA information, and *flag* = 1 information, which are denoted by *N*_*data*_, *N*_*TA*_, and *N*_*flag*=1_, respectively. These are used to evaluate the communication overhead associated with flag information and TA updates by comparing *N*_*TA*_ and *N*_*flag*=1_ with *N*_*data*_. Note that, although these three types of information can be transmitted simultaneously, we count them separately. The following figures show the mean values of each evaluation metric for 10 simulations with a simulation time of 40,000 seconds.

In DC-2d and DC-2d-i, we used *n* = 10. We tried several other values of *n*, but the results did not change significantly.

In the following, we evaluate the continuity and tracking performance of the proposed method using the above metrics and discuss this method’s computational and communication overheads.

#### 4.3.1 Continuity performance.

**Fig 9:**
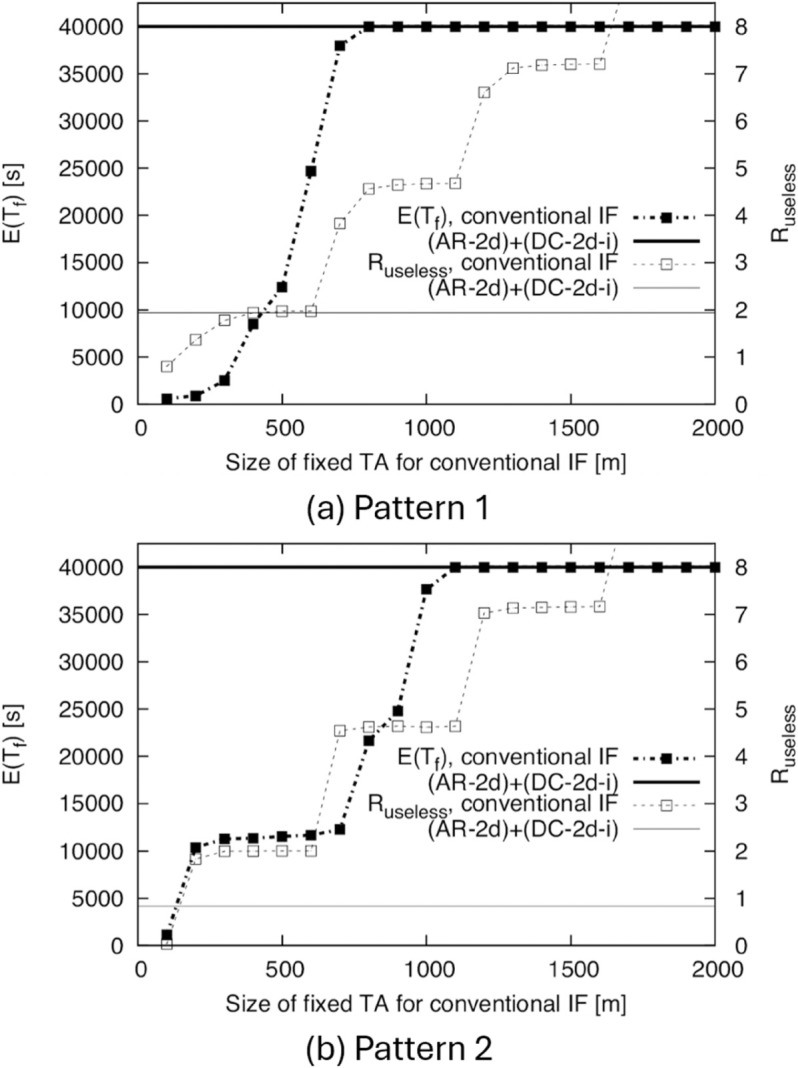
$E(T_{f})$ and $R_{useless}$ for the conventional IF and (AR-2d)+(DC-2d-i) for BF1 to BF5. (a) Pattern 1, (b) Pattern 2.

To evaluate the continuity performance of (AR-2d)+(DC-2d-i) in situations where the density changes, we show the simulation results of *E*(*T*_*f*_) in Fig 9. (AR-2d)+(DC-2d-i) is realized by adding the two functions AR and DC to the conventional IF using a fixed TA. Therefore, to investigate the effects of AR and DC, we also show the results for the conventional IF using a fixed TA in Fig 9 for comparison. Here, we do not compare the proposed method with other IF methods because no IF method has been adapted to the case of time-varying density and the mobility model where nodes never return to the same area. For example, the IF methods proposed in two previous works [18,20] are based on the RWP model, which cannot be used for evaluation of our proposed method as mentioned. Similarly, we do not compare the proposed method with representative protocols for DTNs such as PLOPHET [4] and Spray and Wait [5], since these protocols are designed to deliver information to a specified destination node, and thus their purpose differs from that of IF.

In Fig 9, the horizontal axis is the size of the TA in the conventional IF (distance from O to the edge of the TA). From this figure, in both Patterns 1 and 2, (AR-2d)+(DC-2d-i) achieves a high *E*(*T*_*f*_). In fact, we have *E*(*T*_*f*_) = 40,000 s, which means that the IF never finished completely during the simulation. Note that, in Pattern 2, even though there are time periods when the density is less than the density threshold of λ=0.001099 m^−1^ derived in Sect 4.2, (AR-2d)+(DC-2d-i) successfully prevents the complete termination of IF. This is because the approximation of $\mathrm{Pr}(A_{i})$ used in the theoretical computation is a safe approximation.

On the other hand, in the conventional IF, we can increase *E*(*T*_*f*_) by increasing the size of TA; however, because the size of TA required to achieve *E*(*T*_*f*_) = 40,000 s differs between Patterns 1 and 2, it would be difficult to find the optimal size of TA without prior knowledge of the change in density. The results for *R*_*useless*_ are also shown in Fig 9. Even when using the optimal TA sizes for the conventional IF (800 m for Pattern 1 and 1100 m for Pattern 2), the value of *R*_*useless*_ is significantly larger than for (AR-2d)+(DC-2d-i). From these results, (AR-2d)+(DC-2d-i) successfully prevents the complete termination of IF, and it also significantly reduces unnecessary transmissions compared to the method where the TA is fixed.

#### 4.3.2 Tracking performance.

**Fig 10:**
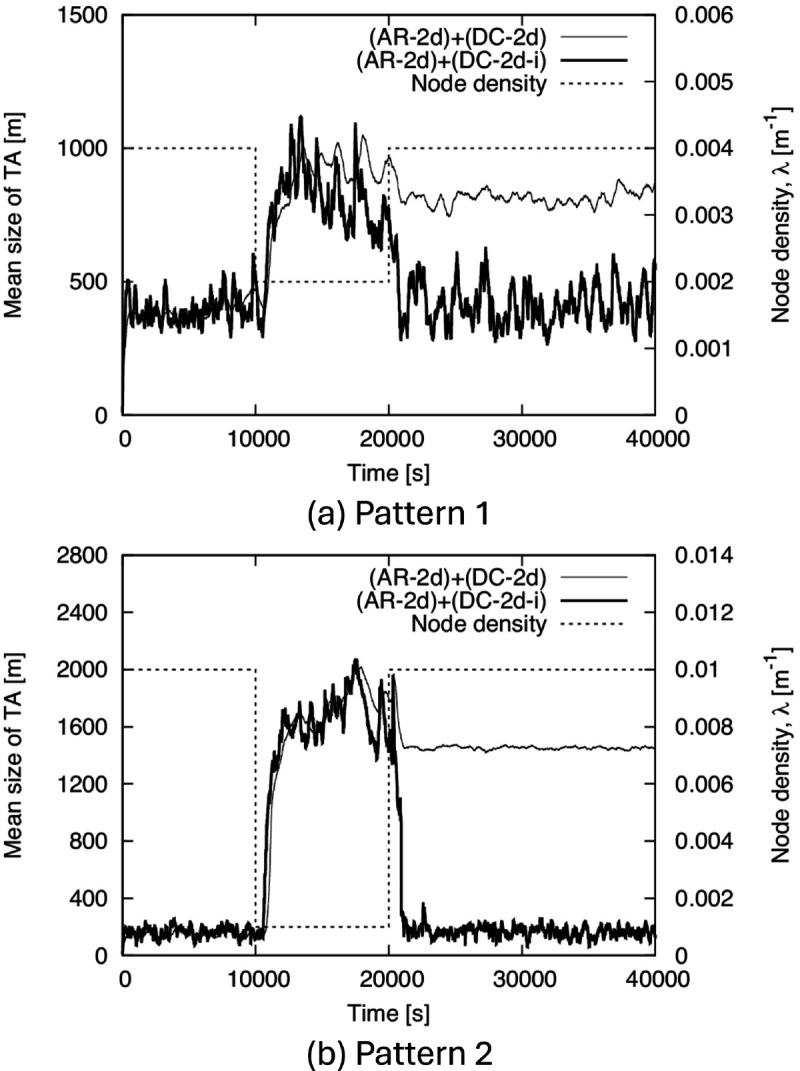
Time variation of the mean size of TA for BF1 to BF5. (a) Pattern 1, (b) Pattern 2.

Next, we show the time-tracking performance of TA size in Fig 10. To confirm the effect of the improvements to the proposed method described in Sect 3.3, we show the results for both (AR-2d)+(DC-2d) and (AR-2d)+(DC-2d-i). From this figure, (AR-2d)+(DC-2d-i) succeeds in increasing and decreasing the TA size in response to the decrease and increase in density, respectively. On the other hand, in (AR-2d)+(DC-2d), the TA size increases in response to the decrease in density but does not sufficiently decrease even when the density increases. This indicates the necessity of introducing a lifetime of the generated TA, as explained in Sect 3.3. In addition, the relationship between the values of *λ* and TA size in (AR-2d)+(DC-2d-i) is roughly within the range estimated in the theoretical evaluation in Sect 4.2.

**Fig 11:**
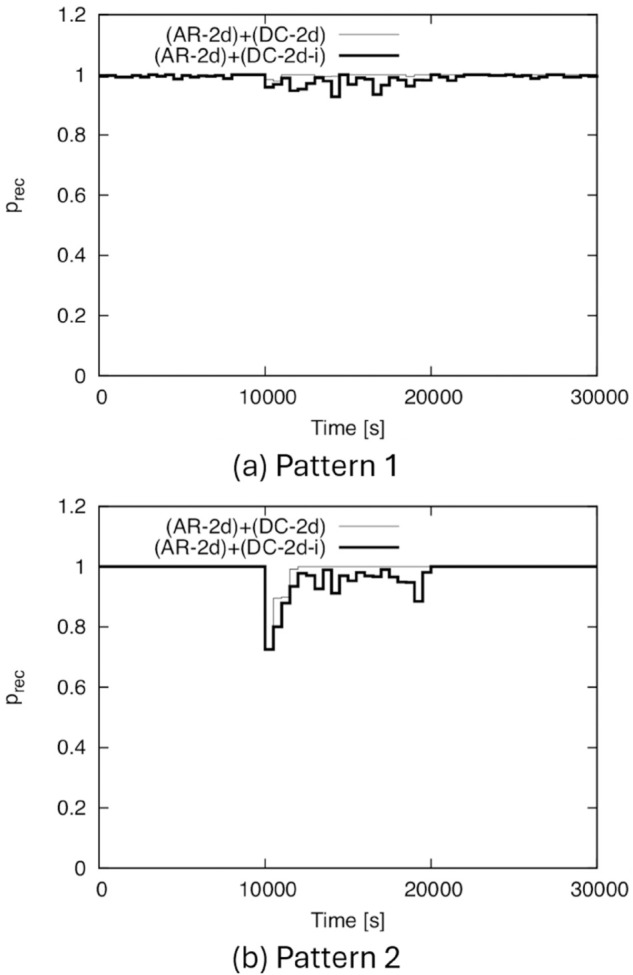
Time variation of $p_{rec}$ for BF1 to BF5. (a) Pattern 1, (b) Pattern 2.

Next, we show the time variation of *p*_*rec*_ in Fig 11. In this figure, we divide the horizontal axis into 500-s intervals and plot the *p*_*rec*_ value as the height of the step for nodes that entered the service area during each time interval. Although the simulation time is 40,000 s, Fig 11 shows the results only up to 30,000 s for readability. From this figure, we can see that in both Patterns 1 and 2, although the density decreases in the middle of the simulation, a high *p*_*rec*_ is always maintained. In Pattern 2, *p*_*rec*_ slightly decreases around 10,000 s. This is because the decrease in density in Pattern 2 is too sudden and significant, causing a slight time lag in the dynamic control of TA for DC-2d and DC-2d-i. Nevertheless, DC-2d and DC-2d-i can track such a sudden change in density, and after about 11,500 s, *p*_*rec*_ returns to nearly 1.

Note that if we apply the conventional IF to Patterns 1 and 2, the performance obviously varies depending on the fixed TA size, but, for example, if we optimize the TA size for the initial density, the IF ends completely just after t=10,000 s due to the sudden reduction in density, and *p*_*rec*_ becomes 0 after this time.

**Fig 12:**
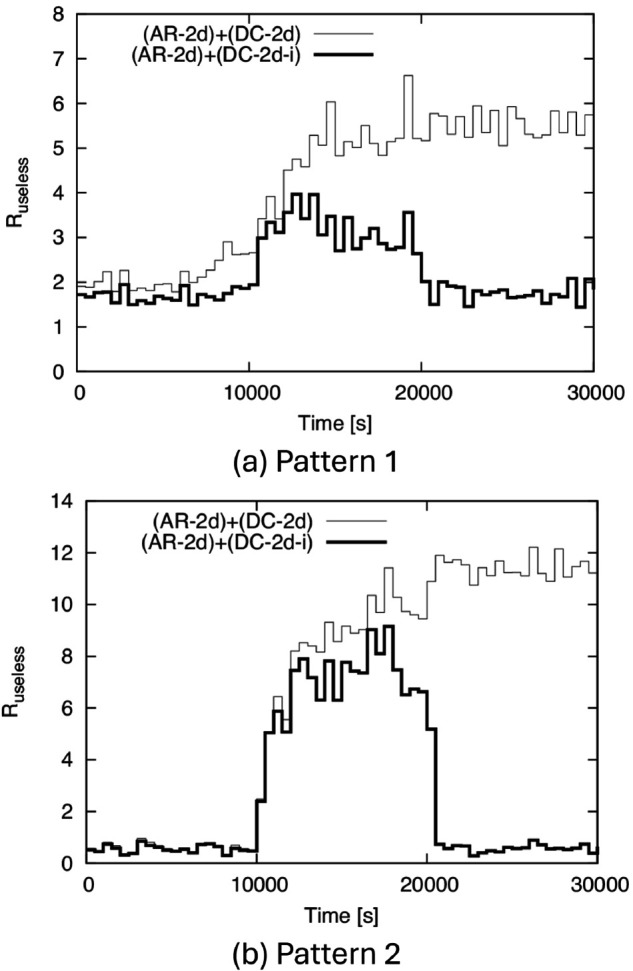
Time variation of $R_{useless}$ for BF1 to BF5. (a) Pattern 1, (b) Pattern 2.

Finally, to observe how the above factors affect useless transmissions, we show the time variation of *R*_*useless*_ in Fig 12. From this figure, *R*_*useless*_ becomes large only between 10,000 s and 20,000 s in (AR-2d)+(DC-2d-i), while it remains large after 20,000 s in (AR-2d)+(DC-2d). This indicates that (AR-2d)+(DC-2d-i) suppresses useless transmissions while continuing IF even when the density changes.

Table 1 shows *p*_*rec*_ and *R*_*useless*_ for (AR-2d)+(DC-2d) and (AR-2d)+(DC-2d-i) over the entire simulation, and we can see that (AR-2d)+(DC-2d-i) keeps *p*_*rec*_ close to 1 while greatly reducing *R*_*useless*_.

**Table 1:** $p_{rec}$ and $R_{useless}$ over the entire simulation for BF1 to BF5.

Density change pattern	Method	$p_{rec}$	$R_{useless}$
Pattern 1	(AR-2d)+(DC-2d)	0.9987	4.380
	(AR-2d)+(DC-2d-i)	0.9918	1.940
Pattern 2	(AR-2d)+(DC-2d)	0.9992	7.539
	(AR-2d)+(DC-2d-i)	0.9978	0.831

#### 4.3.3 Discussion on computational and communication overheads in proposed method.

We also consider the computational load required to execute the proposed method and the communication overhead associated with flag information and TA updates. First, we discuss the computational load. In (AR-2d)+(DC-2d-i), the following processing is required in addition to the conventional IF:

- When a node that already has information receives newer TA information, it updates its own TA information.
- The TA information is reset when the TA’s lifetime expires.

Additionally, the following processing is required only for a node having M with *flag* = 1:

- When the node moves away from O, it sends the M with *flag* = 1 to a node approaching O.
- At that time, it adds its own position to the transmitted position history information and calculates a new TA.

These processes are not complicated, and the total size of the flag and transmitted position history information is extremely small, amounting to at most a few dozen bytes. Therefore, regardless of the network scale, it is considered unlikely that computational load would make practical implementation difficult.

**Fig 13:**
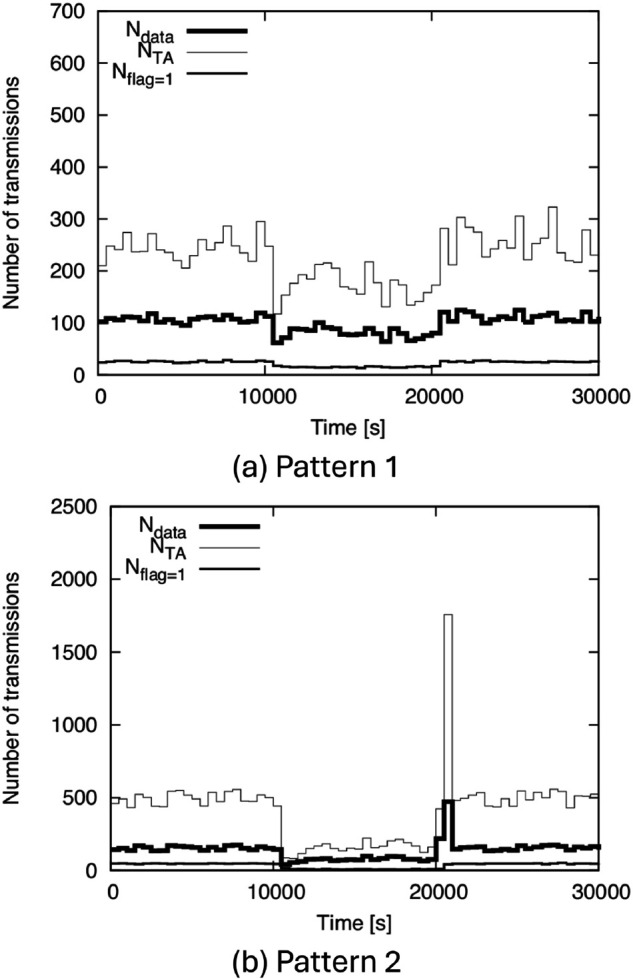
$N_{data}$, $N_{TA}$, and $N_{flag=1}$ for (AR-2d)+(DC-2d-i) with BF1 to BF5. (a) Pattern 1, (b) Pattern 2.

Next, to discuss communication overhead, we show *N*_*data*_, *N*_*TA*_, and *N*_*flag*=1_ for (AR-2d)+(DC-2d-i) in Fig 13. From Fig 13, we can see that *N*_*flag*=1_ is significantly smaller than *N*_*data*_. This can be understood by considering the meaning of *flag* = 1: It is basically sent to a node that is moving to O from outside the TA. Such a node does not yet have the data. This indicates that *flag* = 1 information is transmitted simultaneously with the data in almost all cases. Therefore, regardless of the network scale, *N*_*flag*=1_ is much smaller than *N*_*data*_.

We can also see from Fig 13 that *N*_*TA*_ is approximately twice *N*_*data*_ in many cases. This means that the number of TA update transmissions alone is nearly equal to *N*_*data*_, since a node that receives data also receives TA information simultaneously. The reason for this result is considered as follows. As explained in Sect 3.3, the new TA information is generated at intervals roughly equivalent to how long a node remains within the TA after receiving information. Therefore, a node that receives information will receive updated TA information approximately once before exiting the TA. In some cases, including those involving significantly high density or the moment just after density increases rapidly, *N*_*TA*_ can exceed twice *N*_*data*_. However, even in these cases, *N*_*TA*_ is at most on the order of a few times *N*_*data*_.

From the above discussion, we believe that implementing the proposed method will not impose an unworkable load in terms of either computational or communication overhead, regardless of the network’s scale.

### 4.4 Results with additional factors (AF1 to AF5)

**Fig 14:**
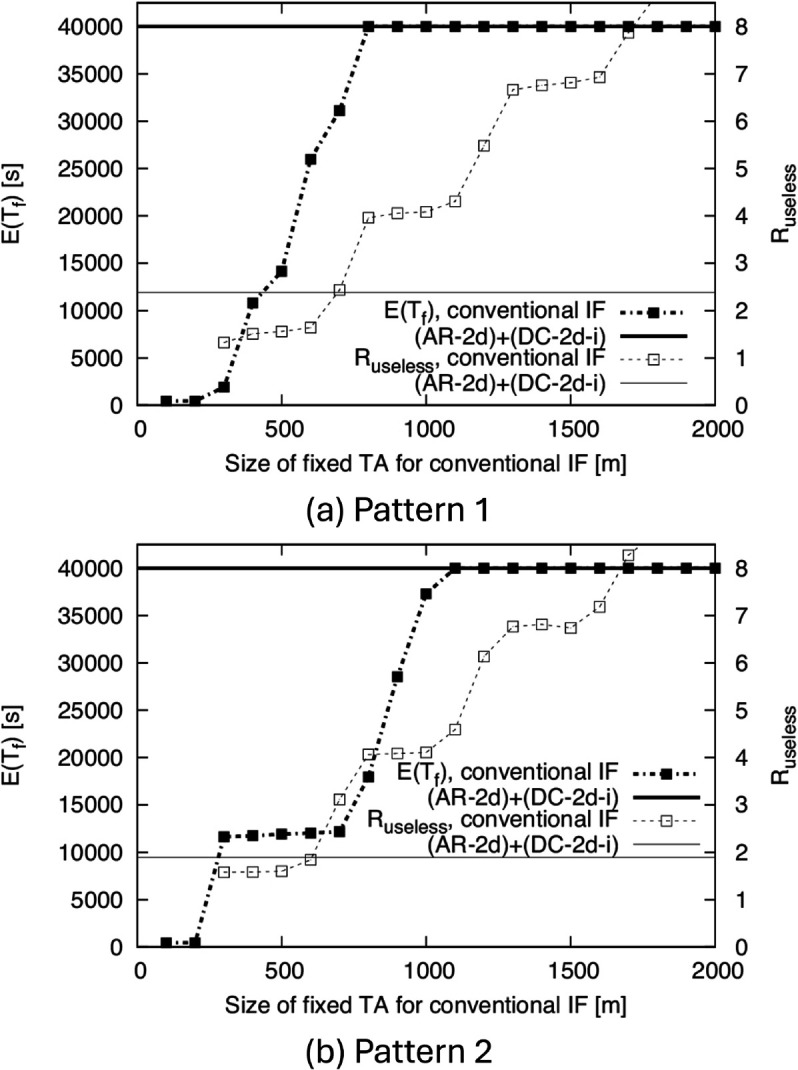
$E(T_{f})$ and $R_{useless}$ for the conventional IF and (AR-2d)+(DC-2d-i) for AF1 to AF5. (a) Pattern 1, (b) Pattern 2.

**Fig 15:**
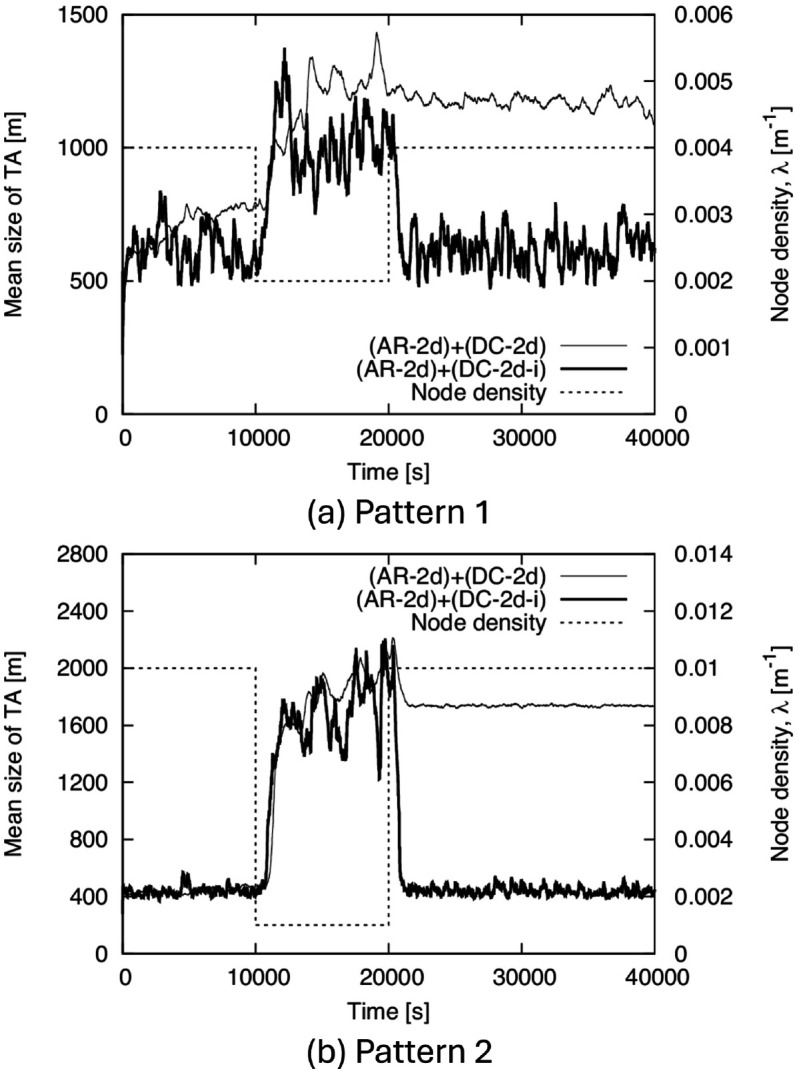
Time variation of the mean size of TA for AF1 to AF5. (a) Pattern 1, (b) Pattern 2.

**Fig 16:**
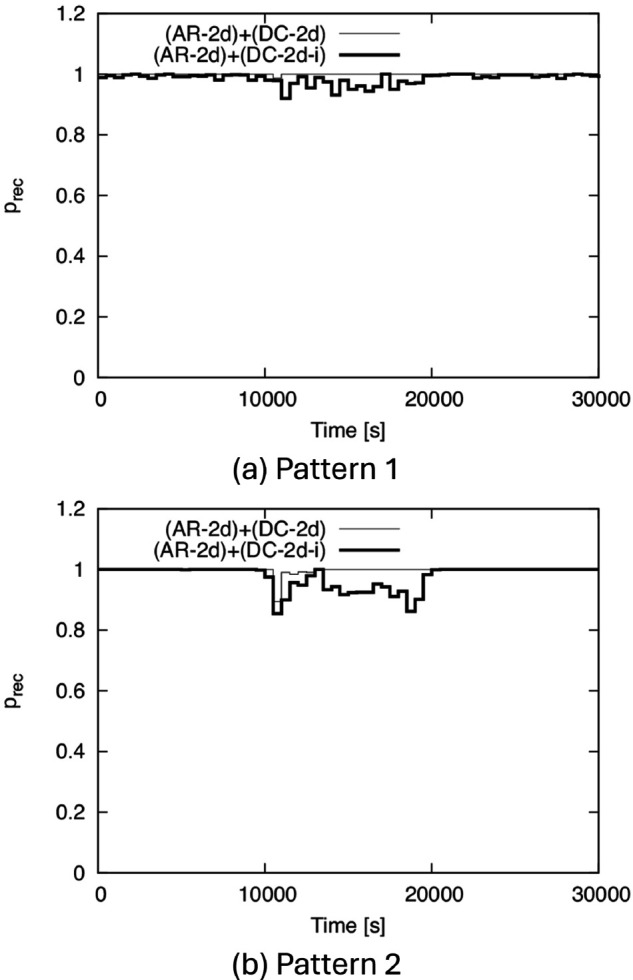
Time variation of $p_{rec}$ for AF1 to AF5. (a) Pattern 1, (b) Pattern 2.

**Fig 17:**
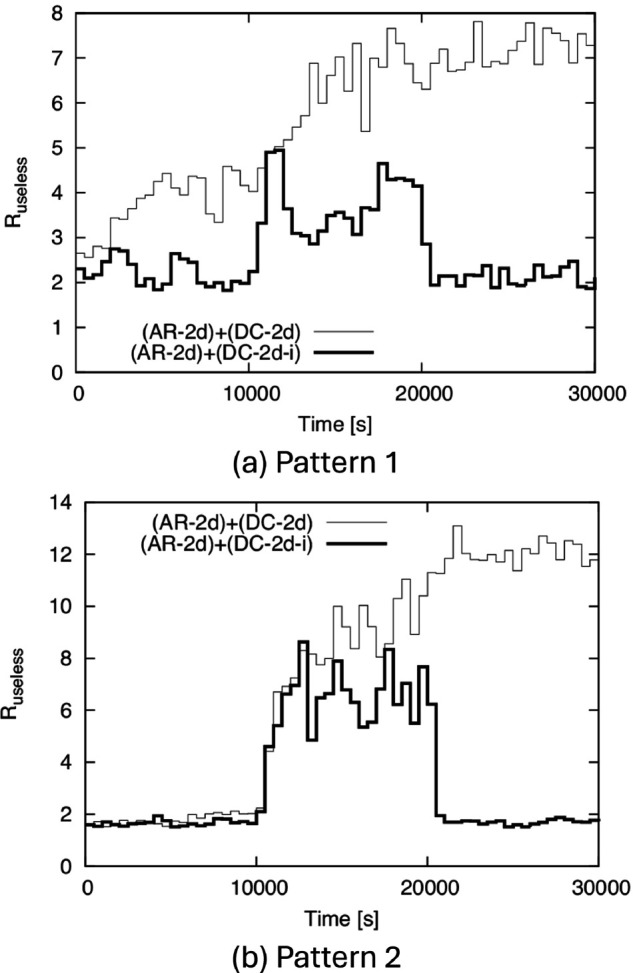
Time variation of $R_{useless}$ for AF1 to AF5. (a) Pattern 1, (b) Pattern 2.

**Fig 18:**
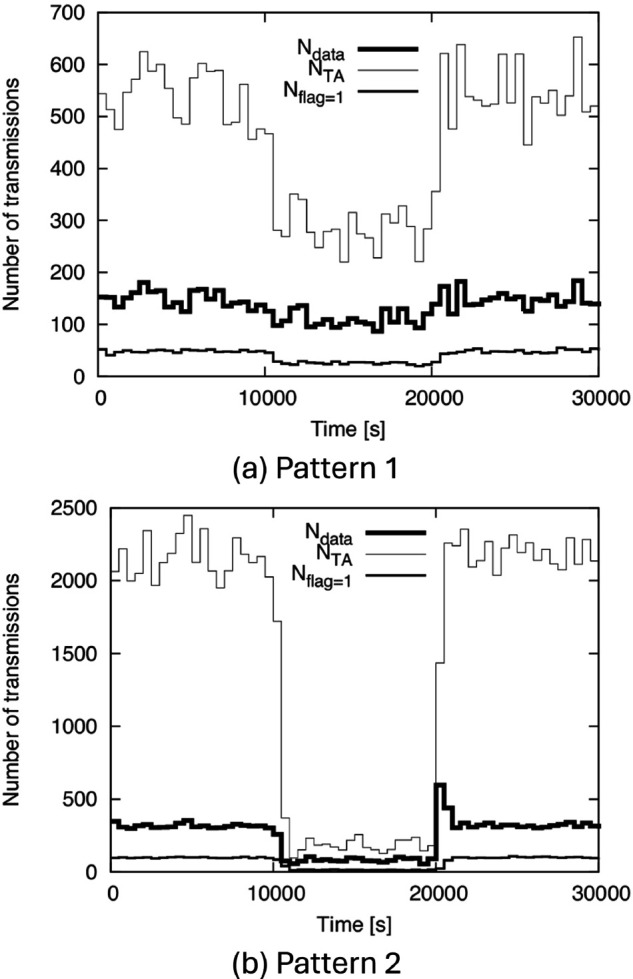
$N_{data}$, $N_{TA}$, and $N_{flag=1}$ for (AR-2d)+(DC-2d-i) with AF1 to AF5. (a) Pattern 1, (b) Pattern 2.

In this subsection, we present simulation results for the proposed method using AF1 to AF5, defined above, to verify its effectiveness even when ideal assumptions are not satisfied. The simulation results are shown in Figs 14 to 18. The evaluation metrics and presentation are the same as in Figs 9 to 13. From Figs 14 to 18, the fundamental trends are largely the same as in the cases for BF1 to BF5 (Figs 9 to 13), although there are some variations in the values. This confirms that (AR-2d)+(DC-2d-i) remains effective even with the additional factors AF1 to AF5.

**Fig 19:**
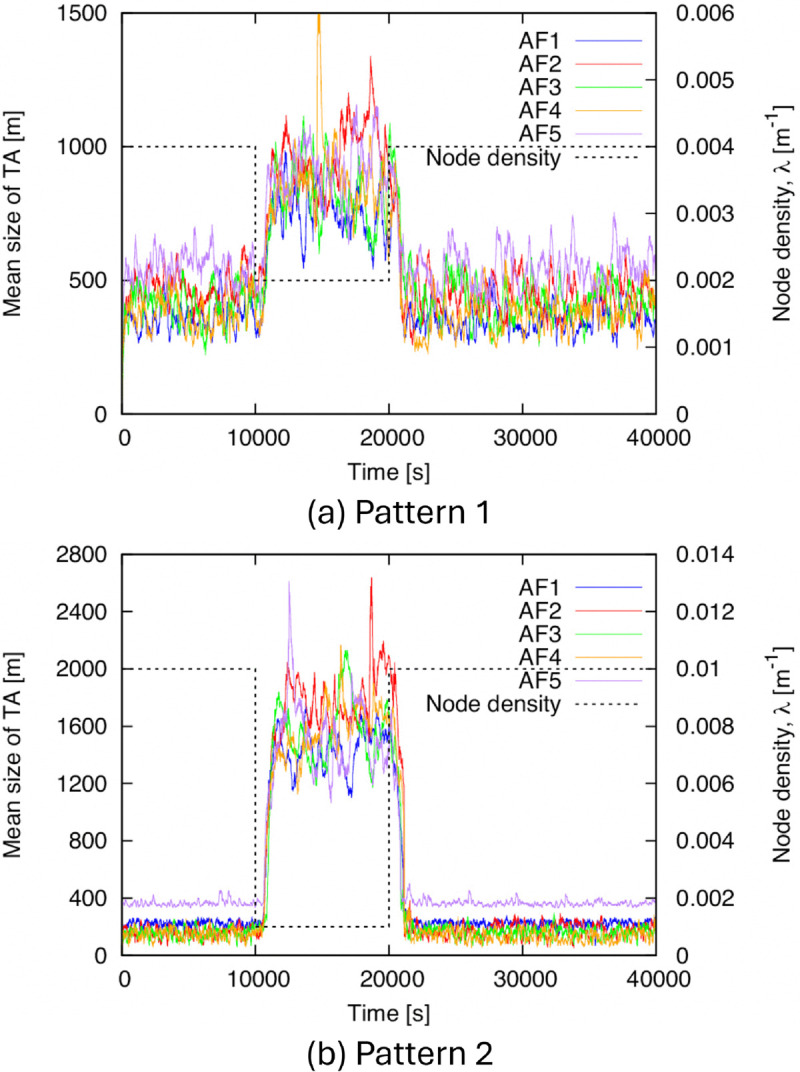
Time variation of the mean size of TA for each factor of AF1 to AF5. (a) Pattern 1, (b) Pattern 2.

Here, to confirm how each factor of AF1 to AF5 influences the results, we show the simulation results for (AR-2d)+(DC-2d-i) when applying each factor individually in Fig 19. In this figure, we specifically show only the time variation of the mean size of TA, since this is a particularly characteristic result. Fig 19 shows that the size of the TA is clearly larger when AF5 is applied than when AF1 to AF4 are applied, particularly during the high-density time intervals of 0 to 10,000 s and 20,000 to 40,000 s. This is assumed to occur because GPS errors cause misjudgments in determining the direction of movement, sometimes resulting in a node moving away from O failing to send M with *flag* = 1 to a node moving toward O. Despite this influence, however, the proposed method successfully prevents the complete termination of IF and achieves superior performance to the fixed TA method, as shown in Figs 14 to 18.

As shown above, the proposed method remains effective even when incorporating factors of variation and randomness within the range of AF1 to AF5. Evaluating and improving the proposed method to account for other factors, such as radio wave interference and irregular road networks, remains a future problem.

## 5 Conclusions

In this paper, we proposed a method to autonomously restart information floating even if it has temporarily terminated in two-dimensional lattice networks. This work was carried out to solve the critical problem of information floating where it is completely terminated. In addition to the autonomous restart method, we also proposed a method to dynamically control the transmittable area for information floating. For the dynamic control method, we emphasized the necessity of setting a lifetime for the generated transmittable area, which improved the proposed method.

The results of theoretical computations and simulations show that the proposed methods can prevent information floating from completely terminating even in situations where the density of mobile nodes decreases suddenly and significantly. In addition, we showed that the dynamic control method can deliver information to almost all of the mobile nodes that need the information, while suppressing unnecessary transmissions.

As mentioned, future work includes conducting evaluations that account for a wider variety of factors, such as radio wave interference, as well as extending the proposed method to road networks of various shapes.

## Appendix

### A. List of acronyms

Table 2 defines the acronyms used in this paper.

**Table 2:** List of acronyms.

Acronym	Full meaning
IF	information floating
TA	transmittable area
AR	autonomous restart
DC	dynamic control of TA
AR-1d	AR for one-dimensional network
DC-1d	DC for one-dimensional network
AR-2d	AR for two-dimensional network
DC-2d	DC for two-dimensional network
DC-2d-i	improved version of DC-2d

### B. Computation of $\mathrm{Pr}(A_{i})$

Since precisely computing $\mathrm{Pr}(A_{i})$ is difficult, we introduce a safe approximation based on two assumptions. First, we assume that information is only transmitted when two nodes pass each other, which means that the effect of the communication range *r* is ignored. Second, we assume that M with *flag* = 1 always returns to O by retracing the intersections it took to move away from O. Under these assumptions, we can easily compute $\mathrm{Pr}(A_{i})$.

First, $\mathrm{Pr}(A_{0})$ can be computed as the probability that a node departed from the left side of O passes another node before reaching intersection C. Therefore, we clearly have

$$\mathrm{Pr}(A_{0})=1-e^{-2a\lambda }.$$ (7)

Next, we compute $\mathrm{Pr}(A_{i})$ for i≥1. This can be expressed as

$$\mathrm{Pr}(A_{i})=\mathrm{Pr}(A_{0})+\{1-\mathrm{Pr}(A_{0})\}\mathrm{Pr}(A_{i}|\overset{―}{A_{0}}),$$ (8)

where $\mathrm{Pr}(A_{i}|\overset{―}{A_{0}})$ is the probability that *A*_*i*_ occurs given that *A*_0_ does not occur (i.e., given that M with *flag* = 1 that enters *s*_1_ from O leaves *s*_1_ without returning to O).

Then, to compute $\mathrm{Pr}(A_{i}|\overset{―}{A_{0}})$, we define *S*_*i*_ as the set of outer road segments of *s*_*i*_ as viewed from O. For example, $S_{1}\;=\;\{s_{2},s_{3},s_{4}\}$, $S_{2}=\{s_{5},s_{6},s_{7}\}$, and $S_{6}=\{s_{17},s_{18},s_{19}\}$. We also define *p*_*pass*_ and *p*_*s*_ as follows.

- *p*_*pass*_: Probability that a node entering *S*_1_ after leaving C will pass another node before leaving *S*_1_. We clearly have $p_{pass}=1-e^{-4a\lambda }$.
- *p*_*s*_: Probability that the above node, moving on *S*_1_ and toward C, will go toward O after reaching C. We clearly have *p*_*s*_ = 1/3.

First, we compute $\mathrm{Pr}(A_{1}|\overset{―}{A_{0}})$. Consider the situation where a node having M with *flag* = 1 (denoted by *n*_0_) enters *S*_1_ after leaving *s*_1_. If *n*_0_ passes another node (denoted by *n*_1_) on a road segment in *S*_1_ (i.e., *s*_2_, *s*_3_, or *s*_4_) and *n*_1_ moves toward *s*_1_ immediately after reaching C, then M with *flag* = 1 will return to O in one step. This probability is computed by $p_{pass}p_{s}$. If *n*_1_ moves to a road segment of *S*_1_ other than *s*_1_ after reaching C, *n*_1_ passes another node (denoted by *n*_2_) on *S*_1_ and *n*_2_ moves toward *s*_1_ after reaching C, then M with *flag* = 1 will return to O in two steps. This probability is computed by $p_{pass}^{2}(1$ − $p_{s})p_{s}$. In general, the probability that M with *flag* = 1 returns to O in *m* steps is computed by $p_{pass}^{m}(1$ − $p_{s}{)}^{m-1}p_{s}$. Therefore, $\mathrm{Pr}(A_{1}|\overset{―}{A_{0}})$ can be computed by adding up these probabilities for m=1,2,⋯ as follows.

$$\mathrm{Pr}(A_{1}|\overset{―}{A_{0}})=\sum_{m=1}^{\infty }p_{pass}^{m}(1-p_{s}{)}^{m-1}p_{s}=\frac{p_{s}p_{pass}}{1-(1-p_{s})p_{pass}}.$$ (9)

Next, we compute $\mathrm{Pr}(A_{2}|\overset{―}{A_{0}})$. The basic idea is the same as the computation of $\mathrm{Pr}(A_{1}|\overset{―}{A_{0}})$, but the probability that an M with *flag* = 1 entering *S*_1_ after leaving C returns to C without leaving *S*_1_ to *S*_4_ is computed as $p_{pass}+(1-p_{pass})\mathrm{Pr}(A_{1}|\overset{―}{A_{0}})$, not *p*_*pass*_. Therefore, $\mathrm{Pr}(A_{2}|\overset{―}{A_{0}})$ is computed by the following equation.

$$\mathrm{Pr}(A_{2}|\overset{―}{A_{0}})=\frac{p_{s}\{p_{pass}+(1-p_{pass})\mathrm{Pr}(A_{1}|\overset{―}{A_{0}})\}}{1-(1-p_{s})\{p_{pass}+(1-p_{pass})\mathrm{Pr}(A_{1}|\overset{―}{A_{0}})\}}.$$ (10)

In the same manner, we generally obtain the following recurrence equation for $\mathrm{Pr}(A_{i}|\overset{―}{A_{0}})$ for i≥1:

$$\mathrm{Pr}(A_{i}|\overset{―}{A_{0}})=\frac{p_{s}\{p_{pass}+(1-p_{pass})\mathrm{Pr}(A_{i-1}|\overset{―}{A_{0}})\}}{1-(1-p_{s})\{p_{pass}+(1-p_{pass})\mathrm{Pr}(A_{i-1}|\overset{―}{A_{0}})\}}.$$ (11)

For clarity, the equations of $\mathrm{Pr}(A_{i}|\overset{―}{A_{0}})$ are summarized in Table 3.

**Table 3:** Equations of $\mathrm{Pr}(A_{i}|\overset{―}{A_{0}})$.

*i* = 1	$\mathrm{Pr}(A_{1}|\overset{―}{A_{0}})=\frac{p_{s}p_{pass}}{1-(1-p_{s})p_{pass}}$
*i* = 2	$\mathrm{Pr}(A_{2}|\overset{―}{A_{0}})=\frac{p_{s}\{p_{pass}+(1-p_{pass})\mathrm{Pr}(A_{1}|\overset{―}{A_{0}})\}}{1-(1-p_{s})\{p_{pass}+(1-p_{pass})\mathrm{Pr}(A_{1}|\overset{―}{A_{0}})\}}=\frac{p_{pass}p_{s}\{p_{s}^{2}-(1-p_{pass}{)}^{2}\}}{p_{pass}p_{s}^{3}-(1-p_{pass}{)}^{3}(1-p_{s})}$
*i* = 3	$\mathrm{Pr}(A_{3}|\overset{―}{A_{0}})=\frac{p_{s}\{p_{pass}+(1-p_{pass})\mathrm{Pr}(A_{2}|\overset{―}{A_{0}})\}}{1-(1-p_{s})\{p_{pass}+(1-p_{pass})\mathrm{Pr}(A_{2}|\overset{―}{A_{0}})\}}=\frac{p_{pass}p_{s}\{p_{s}^{3}-(1-p_{pass}{)}^{3}\}}{p_{pass}p_{s}^{4}-(1-p_{pass}{)}^{4}(1-p_{s})}$
⋮	⋮
*i*	$\mathrm{Pr}(A_{i}|\overset{―}{A_{0}})=\frac{p_{s}\{p_{pass}+(1-p_{pass})\mathrm{Pr}(A_{i-1}|\overset{―}{A_{0}})\}}{1-(1-p_{s})\{p_{pass}+(1-p_{pass})\mathrm{Pr}(A_{i-1}|\overset{―}{A_{0}})\}}=\frac{p_{pass}p_{s}\{p_{s}^{i}-(1-p_{pass}{)}^{i}\}}{p_{pass}p_{s}^{i+1}-(1-p_{pass}{)}^{i+1}(1-p_{s})}$

By solving this recurrence equation and substituting the solution of $\mathrm{Pr}(A_{i}|\overset{―}{A_{0}})$ into Eq (8), we have the general solution of $\mathrm{Pr}(A_{i})$ as follows.

$$\mathrm{Pr}(A_{i})=1+\frac{e^{-2a\lambda }(1-p_{pass}{)}^{i}(1-p_{s}{)}^{i}(p_{pass}+p_{s}-1)}{(1-p_{pass}{)}^{i+1}(1-p_{s}{)}^{i+1}-p_{pass}p_{s}^{i+1}(1-p_{s}{)}^{i}}.$$ (12)

Furthermore, we can compute $\lim_{i\to \infty }\mathrm{Pr}(A_{i})$, which means the probability that M with *flag* = 1 eventually returns to O despite how far it has travelled, as follows.

$$\lim_{i\to \infty }\mathrm{Pr}(A_{i})=\left\{ \begin{array}{cc} \frac{2-3e^{-2a\lambda }+e^{2a\lambda }}{2}, & \lambda <\frac{\log3}{4a},\\ 1, & \text{otherwise.} \end{array}\right.$$ (13)
